# Time Course of Metabolic Capacities in Paralarvae of the Common Octopus, *Octopus vulgaris*, in the First Stages of Life. Searching Biomarkers of Nutritional Imbalance

**DOI:** 10.3389/fphys.2017.00427

**Published:** 2017-06-16

**Authors:** Amalia E. Morales, Gabriel Cardenete, M. Carmen Hidalgo, Diego Garrido, M. Virginia Martín, Eduardo Almansa

**Affiliations:** ^1^Departamento de Zoología, Facultad de Ciencias, Universidad de GranadaGranada, Spain; ^2^Centro Oceanográfico de Canarias, Instituto Español de OceanografíaSanta Cruz de Tenerife, Spain

**Keywords:** *Octopus vulgaris*, paralarvae, metabolic organization, nutritional imbalance, biomarkers

## Abstract

The culture of the common octopus (*Octopus vulgaris*) is promising since the species has a relatively short lifecycle, rapid growth, and high food conversion ratios. However, recent attempts at successful paralarvae culture have failed due to slow growth and high mortality rates. Establishing an optimal nutritional regime for the paralarvae seems to be the impeding step in successful culture methods. Gaining a thorough knowledge of food regulation and assimilation is essential for paralarvae survival and longevity under culture conditions. The aim of this study, then, was to elucidate the characteristic metabolic organization of octopus paralarvae throughout an ontogenic period of 12 days post-hatching, as well as assess the effect of diet enrichment with live prey containing abundant marine phospholipids. Our results showed that throughout the ontogenic period studied, an increase in anaerobic metabolism took place largely due to an increased dependence of paralarvae on exogenous food. Our studies showed that this activity was supported by octopine dehydrogenase activity, with a less significant contribution of lactate dehydrogenase activity. Regarding aerobic metabolism, the use of amino acids was maintained for the duration of the experiment. Our studies also showed a significant increase in the rate of oxidation of fatty acids from 6 days after-hatching. A low, although sustained, capacity for *de novo* synthesis of glucose from amino acids and glycerol was also observed. Regardless of the composition of the food, glycerol kinase activity significantly increased a few days prior to a massive mortality event. This could be related to a metabolic imbalance in the redox state responsible for the high mortality. Thus, glycerol kinase might be used as an effective nutritional and welfare biomarker. The studies in this report also revealed the important finding that feeding larvae with phospholipid-enriched *Artemia* improved animal viability and welfare, significantly increasing the rate of survival and growth of paralarvae.

## Introduction

Commercial octopus fishing has been exploited for several decades leading to strict regulations on fishing practices. As a result, attempts have been made to culture *Octopus vulgaris* in captivity. These practices remain promising since the species has a relatively short lifecycle, fast growth, and high food conversion ratios. However, under culture conditions, paralarvae of this cephalopod species show slow growth and high mortality rates, reflecting the main obstacle to successful culture. Nutritional deficiencies and food source imbalances are considered the primary causes for low paralarvae survival and growth, although other factors related to zootechnical conditions (such as, tank volume, culture density, and light) cannot be ruled out (Iglesias and Fuentes, 2014).

In terms of nutrition, Lee (1994) highlighted the importance of closely balanced amino acid levels in larval food needed as substrates for metabolism and protein synthesis. On the other hand, Navarro et al. (2014) reported that a nutritional imbalance in both content and profile of the fatty acid in artificial food sources may be responsible for high mortalities since reared paralarvae differ from recently hatched individuals especially in these aspects. Establishing an optimal nutritional regime is of paramount importance for successful thriving of paralarvae and efficient aquaculture. Thus, it is essential to have a thorough knowledge of the physiological processes regulating food assimilation and metabolism.

Regarding metabolic capabilities of cephalopods, studies have shown predominance in protein catabolism, regardless of individual body mass (Boucher-Rodoni and Mangold, 1985; Lee, 1994; Katsanevakis et al., 2005; Petza et al., 2006). This implies a high requirement of protein in available food sources. Additionally, a low capacity to use lipids as metabolic fuel has been reported throughout the cephalopod literature (Ballantyne et al., 1981; Storey and Storey, 1983; O'Dor and Webber, 1986; Lee, 1994; Hochachka, 1995).

After the larval yolk reserves are exhausted, paralarvae depend solely on exogenous food. Octopuses in this stage survive on live prey, which are actively captured using bursts of anaerobic swimming (Baldwin, 1982). In vertebrates, energy needed for early stage activity is supported by the creatine phosphate/creatine kinase ATP regeneration system, followed by the pyruvate fermentation by lactate dehydrogenase (LDH). Interestingly, in mollusks, anaerobic conditions, in addition to the LDH activity, have been shown to produce energy by the arginine phosphate/arginine kinase system in which octopine dehydrogenase (ODH) is involved (Lyzlova and Stefanov, 1991). In many species of mollusks only ODH activity is present; when both ODH and LDH are operating, ODH exhibits the higher activity (Regnouf and van Thoai, 1970; Gäde, 1980; Speers-Roesch et al., 2016). Regarding the advantage of ODH over LDH, Fields and Quinn (1981) reported that ODH maintains a lower cytosolic redox ratio (NADH/NAD^+^) than LDH during anoxia, where the glycolytic pathway is prevalent.

Although metabolic capacities in adult cephalopods have been assessed in several studies (O'Dor and Wells, 1987; Lee, 1994), such studies have not been reported for early stage larvae. Thus, this report seeks to elucidate the metabolic organization in common octopus paralarvae throughout ontogenic development and to assess the capacity of adaptations to changes in food composition.

## Materials and methods

All experimental work was performed according to Spanish law (RD 53/2013) based on the European Union's directive on animal welfare for the protection of animals used for scientific purposes (Directive 2010/63/EU). Guidelines for the care and welfare of cephalopods proposed by Fiorito et al. (2015) were followed in this study. The present study was also approved (register document CEIBA2014-0108) by the Ethics Committee for Animal Research and Welfare (Comité de Ética de la Investigación y Bienestar Animal, CEIBA) from the University of La Laguna (Spain).

### Paralarvae rearing conditions

Two experiments were carried out to characterize the metabolic profile in common octopus paralarvae. The first experiment analyzed the time course of metabolic capacities during the first 12 days of life. The second experiment analyzed the influence of food composition (with a diet rich in highly unsaturated fatty acids and phospholipids) on metabolic capacities and survival.

To carry out both experiments, a total of 20 adult *Octopus vulgaris* were captured by local fishermen using artisanal octopus traps in Tenerife coastal waters (Canary Islands, Spain) and maintained in the facilities of the Oceanographic Centre of the Canary Islands (Spanish Institute of Oceanography). Adult specimens were kept in 1,000 L tanks (with a maximum density of 10 kg/tank) with water renovation (5 L/min), under oxygen saturation conditions and low light intensity. Two batches of paralarvae obtained from this broodstock were used in the experiment described below.

### Experiment 1. time course of metabolic capacities during the first 12 days of life

A total of 15,000 paralarvae, (5,000 paralarvae/tank; 5 paralarvae /L), were reared in triplicate during 12 days in 1,000 L black fiberglass cylinder-conical tanks with a flow-through seawater system at 60 mL/s from 18:00 to 8:00 (over 2.5 renewals/day). The renovation flow allowed the unfed *Artemia* to go through a 500 μm outflow mesh located in the middle of the tank. Moderated flux aeration stones were placed on the edge of the tanks. Green water (1 × 10^6^ cell/mL *Nannochloropsis* sp supplied by Phytobloom Green Formula®, Olhão, Portugal) was added at 8:00. The natural photoperiod was attained at a maximum intensity (around mid-day) of 300 lx. Temperature and oxygen were measured daily, and nitrite, ammonium, and salinity once a week (see Table 1). Paralarvae were fed with *Artemia* (Sep-Art BF INVE Aquaculture, Dendermonde, Belgium) enriched for 20 h after hatching with freeze dried *Isochrysis galbana* (supplied by easy algae®, Cádiz, Spain; 10 metanauplii/mL, 1·10^7^ cell/mL). *Artemia* was supplied at 0.3 *Artemia*/mL divided in three times a day (at 10:00, 13:00, and 16:00). In order to avoid enrichment lost, *Artemia* cultures were kept in the dark at 4°C with soft aeration until the moment of feeding.

**Table 1:** Physicochemical parameters for paralarvae reared.

	**Experiment 1**	**Experiment 2**
T (° C)	22.3 ± 0.5	22.57 ± 0.1
0_2_ (%)	91.8 ± 0.8	94.50 ± 1.0
Salinity (psu)	36.8 ± 0.1	36.8 ± 0.1
pH	8.1 ± 0.0	8.1 ± 0.0
N0${}_{2}^{-}$ (mg/L)	<0.3	<0.3
NH${}_{4}^{+}$ (mg/L)	0.0	0.0

*Data are shown as mean ± standard deviation*.

### Experiment 2. influence of food composition on metabolic capacities and survival

A total of 30,000 paralarvae, (5,000 paralarvae per tank; 10 paralarvae/L) were reared over 28 days in 500 L black fiberglass cylinder-conical tanks. Two fluorescent lights (OSRAM Dulux superstar 36W/840) were placed above each tank to attain 700 lx focused in the middle of the tank surface with a 12L:12D photoperiod (8:00–20:00). A flow-through seawater system equipped with 20, 5, and 1 μm filter cartridges and UV lamps was used. A water flow per tank of 1 L/min (over 1.5 renewals/day) was applied from 18:00 to 8:00. In similar protocol to the first experiment, the renovation flow allowed the unfed *Artemia* to go through a 500 μm outflow mesh located in the middle of the tanks. Two moderated flux aeration stones were placed in front each other in the edges of the tanks. Green-water system using 5 × 10^5^ cell/mL of *Nannochloropsis sp* (Phytobloom Green Formula®, Olhão, Portugal) was added to the tanks before illumination. Temperature and oxygen were measured daily, and nitrite, ammonium and salinity once a week (see Table 1).

Paralarvae were fed with the same *Artemia* used in the first experiment, but in this case, the control group (C) was enriched with microalgae (freezer dried *Isochrysis galbana*, and *Nannochloropsis sp*) or with an experimental diet of Marine Lecithin LC 60® (PhosphoTech Laboratoires, Saint Herblain, France; LC60). Each treatment was carried out in triplicate. To quantitate *Artemia* size over the duration of the experimental period, three prey sizes were used: first, nauplii from day 0 to 3; second, metanauplii from day 4 to 11; and third, 8 day old metanauplii from day 12 to 27. Enrichments and on-growing of *Artemia* were made according to Garrido et al. (2017). *Artemia* was supplied at 0.5 *Artemia*/mL divided in three times a day (at 10:00, 13:00, and 16:00). In order to avoid enrichment loss, *Artemia* cultures were kept in the dark at 4°C with soft aeration until the moment of feeding.

### Growth and survival

No dry weight was determined in Experiment 1. In the second experiment, 15 individuals' dry weight (DW) was determined for each treatment at day 0, 12, and 28. Paralarvae were euthanized in chilled seawater (−2°C), washed in distilled water, oven dried (110°C, 20 h) and weighed. Specific growth rate (SGR, % DW/day) was calculated as (Ln DWf-Ln DWi) 100/(tf-ti), where DWf and DWi are the dry weight at final time (tf) and initial time (ti) respectively. Survival was assessed in both experiments at the termination of the experiment. Survival (S, %) was calculated as S = 100 Xf/(Xi-Xs), where Xf is the number of live individuals at the end of experiment, Xi is the initial number of individuals and Xs is the number of individuals sacrificed during the experiment.

### Sample collection

Due to the usual mortality in this type of culture and the large size of samples that require analyses, sampling could only be extended up to 12 days. Thus, sample collections in experiment 1 were carried out at 0, 3, 6, 9, and 12 days.

Likewise in the second experiment, samples were taken at 12 days from C and LC groups, and also at 28 days in LC groups since this treatment showed greater survival allowing for a suitable sample size.

Pools of 300 paralarvae per tank were taken in each sampling. Paralarvae were euthanized using ice seawater (−2°C), frozen in liquid nitrogen and stored at −80°C until further analysis.

### Enzyme assays

Pooled paralarvae of each sample were homogenized in four volumes of ice-cold 100 mM Tris-HCl buffer containing 0.1 mM EDTA and 0.1% (v/v) Triton X-100, pH 7.8. All procedures were performed on ice. Homogenates were centrifuged at 30,000 × g for 30 min at 4°C and the resultant supernatants were kept in aliquots and stored at −80°C for further enzyme assays.

All enzyme assays were performed at 25°C using a PowerWaveX microplate scanning spectrophotometer (Bio-Tek Instruments, Inc., USA) and run in duplicate in 96-well microplates (UVStar Greiner Bio-One, Germany). The optimal substrate and protein concentrations for the measurement of maximal activity for each enzyme in each tissue were established by preliminary assays. The millimolar extinction coefficients used for NADH/NADPH and DTNB, were 6.22 and 13.6 mM^−1^ cm^−1^, respectively.

Activities of fructose 1,6-bisphosphatase (FBPase; EC 3.1.3.11), glycerol kinase (GyK; EC 2.7.1.30), pyruvate kinase (PK, EC 2.7.1.40), glucose-6-phosphate dehydrogenase (G6PDH; EC 1.1.1.49), citrate synthase (CS; EC 4.1.3.7), β-hydroxyacyl CoA dehydrogenase (HOAD; EC 1.1.1.35), glutamate pyruvate transaminase (GPT; EC 2.6.1.2), glutamate oxaloacetate transaminase (GOT; EC 2.6.1.1), and glutamate dehydrogenase (GDH; EC 1.4.1.2) were determined as previously described by Hidalgo et al. (2017). Octopine dehydrogenase (ODH; EC 1.5.1.11), and lactate dehydrogenase (LDH, EC 1.1.1.27) were assayed according to the method of Baldwin and England (1980). See Supplementary Material provided for detailed assay conditions in a final volume of 200 microliters.

Soluble protein concentration in homogenates was analyzed using the method of Bradford (1976), with bovine serum albumin used as standard.

### Data analysis and statistic

Data were checked for normal distribution with one-sample Kolmogorov-Smirnoff test, as well as for homogeneity of variances with the Levene's test (Zar, 1999) and transformed (natural logarithm) when needed (Fowler et al., 1998). Differences between two groups were assessed by Student's *t*-test. Multiple comparisons in experiment 1 were performed by mean of one-way ANOVA test and Tukey's HSD post hoc test. When normal distribution and/or homoscedasticity were not achieved, data were subjected to Kruskall–Wallis non-parametric test, followed by Games-Howell non-parametric multiple comparison test (Zar, 1999). Statistical significance was established at *P* < 0.05. Statistical analyses were performed using the SPSS package version 15.0 (SPSS Inc., Chicago, USA).

## Results

Table 2 shows the time course of metabolic capacities during the first 12 days of life: newly hatched (0), 3, 6, 9, and 12 day old paralarvae (Experiment 1).

**Table 2:** Ontogenic changes in the activity of key enzymes of intermediary metabolism in *Octopus vulgaris* palarvae.

**Day**	**FBPase**	**GyK**	**PK**	**LDH**	**ODH**	**G6PDH**	**CS**	**HOAD**	**GPT**	**GOT**	**GDH**
**0**	0.8 ± 0.0^ab^	49.3 ± 5.0^b^	126.1 ± 13.2	30.4 ± 0.6^ab^	46.7 ± 6.9^a^	8.2 ± 2.6	59.6 ± 7.1	5.0 ± 0.6^a^	10.5 ± 0.7	201.2 ± 29.9 ^a^	17.1 ± 1.2^a^
**3**	0.6 ± 0.0^a^	37.4 ± 8.1^ab^	108.6 ± 5.8	40.4 ± 1.6^bc^	41.5 ± 5.0^a^	11.4 ± 0.9	81.2 ± 1.2	6.5 ± 0.8^a^	8.3 ± 1.1	272.3 ± 5.8 ^b^	21.4 ± 2.0^a^
**6**	1.2 ± 0.1^b^	29.9 ± 1.0^a^	131.0 ± 8.8	45.0 ± 3.7^c^	51.6 ± 8.0^a^	13.2 ± 1.1	74.1 ± 7.2	16.2 ± 4.3^b^	8.5 ± 0.2	262.1 ± 0.8 ^b^	29.9 ± 2.6^a^
**9**	1.2 ± 0.2^b^	37.9 ± 2.2^ab^	113.0 ± 7.7	34.8 ± 2.1^abc^	73.9 ± 8.0^ab^	12.3 ± 1.2	72.3 ± 2.1	13.5 ± 1.5^b^	10.2 ± 0.4	261.8 ± 12.5 ^b^	44.0 ± 3.7^b^
**12**	1.1 ± 0.2^b^	36.8 ± 3.8^ab^	106.0 ± 4.9	27.2 ± 3.9^a^	95.2 ± 7.4^b^	12.4 ± 0.8	67.2 ± 4.5	12.6 ± 0.9^b^	7.5 ± 0.4	223.1 ± 12.6^ab^	27.7 ± 3.7^a^

*Values are mean ± S.E. (n = 3). Enzymatic activities are expressed as mU mg protein^−1^. Different letters in the same column indicate significant differences (P < 0.05)*.

The PK (glycolysis), G6PDH (NADPH provision), CS (oxidative metabolism), and GPT (amino acid catabolism) activities did not show significant changes during the period analyzed. Nevertheless, FBPase activity decrease at day 3, to subsequently increase and remain unchanged until the end of the experimental period.

Activity of enzymes involved in anaerobic metabolism revealed that LDH reached maximum activity on day 6, after decreasing to values similar to the 0 day. ODH activity increased progressively from day 3 of life, reaching a significant maximum at 12 days old.

Activity of β-oxidation of fatty acids (HOAD) showed a significant increase from day 6 onwards. Enzymes involved in protein metabolism, specifically, the activity of GOT, showed significant increase from day 3 post-hatching, remaining significantly higher until day 9. However, at day 12 the activity was similar to that of day 0. In turn, GDH activity increased gradually, reaching statistical significance at day 9 post-hatching. Again on day 12, activity significantly decreased.

**Figure 1:**
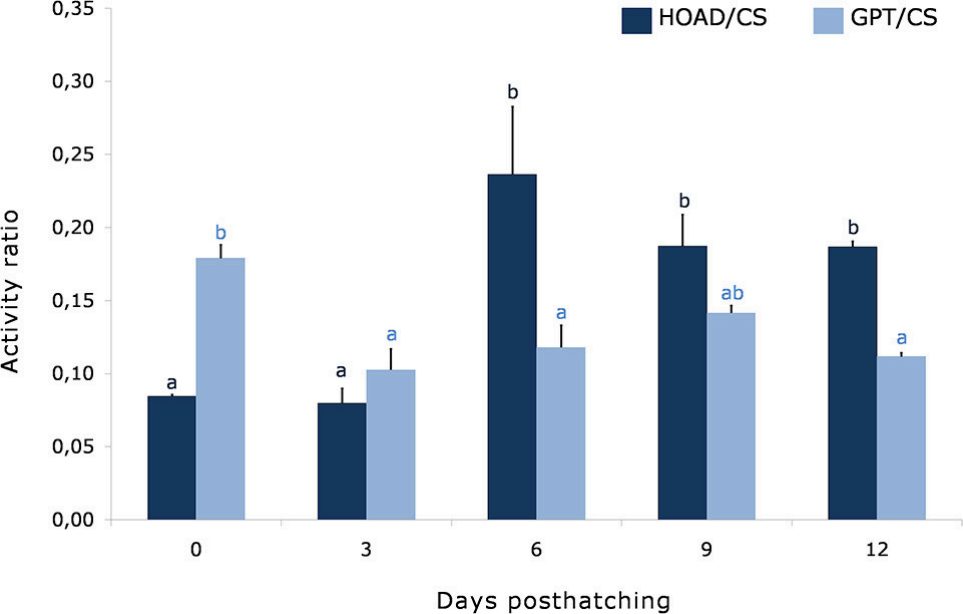
Time course of the ratio between glutamate pyruvate transaminase and citrate synthase (GPT/CS), and β-hydroxyacyl CoA dehydrogenase and citrate synthase (HOAD/CS) activities in octopus paralarvae from Experiment 1. Different superscripts indicate significant differences between sampling points for each parameter (*n* = 3).

The HOAD/CS ratio increased since the sixth day forward, and the GPT/CS ratio was higher in newly hatched paralarvae and decreased from the third day onwards (Figure 1).

In experiment 2, the effect of feeding with *Artemia* enriched with marine phospholipids on metabolic enzyme activity of hatchlings, 12 day and 28 day old paralarvae was studied. Table 3 shows the results of dry weight (DW), specific growth rate (SGR), and survival (S) at the hatchling stage, 12 day, and 28 day-old paralarvae fed with *Artemia* enriched with phytoplankton (Control Diet) or *Artemia* enriched with Marine Lecithin (LC diet). The results showed that DW was significantly higher in paralarvae from the LC group compared to the control group at 12 days old (*P* < 0.05). Also, survival in 28 day old paralarvae was significantly higher in the LC group compared to the control group (*P* < 0.05).

**Table 3:** Dry weight (DW), specific growth rate (SGR), and survival (S) in common octopus paralarvae at hatching and reared for 12 and 28 days with control diet (C, *Artemia* enriched with phytoplankton) or LC diet (*Artemia* enriched with Marine Lecithin).

	**Hatchlings**	**12 days**		**28 days**	
		**12C**	**12LC**	**28C**	**28LC**
**DW (mg)**	0.23 ± 0.03	0.30 ± 0.04	0.36 ± 0.06 ^*^	0.50 ± 0.10	0.57 ± 0.16
**SGR (%)**		2.08	3.59	2.77	3.24
**S (%)**				1.50 ± 1.28	11.70 ± 3.41^*^

Data are presented as mean ± SD. n = 15 for SGR, n = 3 for S.

(*) *Indicate significant differences between C and LC groups (P < 0.05)*.

Table 4 shows the results of metabolic enzyme activities in newly hatched paralarvae, 12 day old paralarvae fed on microalgae-enriched (12C) and on LC60-enriched (12LC) *Artemia*, and in 28 day old paralarvae fed on LC60-enriched *Artemia* (28LC). Of these activities, ODH, CS, HOAD, and GOT increased significantly in 12 day old paralarvae. The only difference among 12C and 12LC paralarvae was the significantly higher GyK activity in 12C. The 28 day old paralarvae fed on LC60-enriched *Artemia* (28LC) showed significant increases in GyK, PK, G6PDH, LDH, GOT, and GDH activities with respect to 12LC.

**Table 4:** Activity of key enzymes of intermediary metabolism in *Octopus vulgaris* palarvae at hatching (0) and after 12 and 28 days of feeding with control diet (C, *Artemia* enriched with phytoplankton) or LC60 diet (*Artemia* enriched with Marine Lecithin).

	**0**	**C Diet**	**LC 60 Diet**	
		**12C**	**12LC**	**28LC**
FBPase	0.9 ± 0.2	0.6 ± 0.1	0.9 ± 0.1	1.2 ± 0.1
GyK	44.2 ± 1.8	58.5 ± 2.4^#^	40.9 ± 3.5	88.9 ± 5.6^a^
PK	123.9 ± 6.7	133.1 ± 9.3	155.6 ± 7.8	175.9 ± 12.7^a^
LDH	24.7 ± 1.2	23.1 ± 1.3	23.2 ± 1.6	33.1 ± 2.5^a^
ODH	37.2 ± 1.1^*^	80.9 ± 8.2	78.9 ± 7.7	62.6 ± 8.8
G6PDH	12.2 ± 0.6	12.8 ± 0.1	11.2 ± 1.7	17.8 ± 0.7^a^
CS	53.0 ± 3.3^*^	87.2 ± 7.5	80.6 ± 5.8	87.4 ± 3.5
HOAD	6.2 ± 0.2^*^	10.7 ± 1.9	12.0 ± 1.5	14.0 ± 1.0
GPT	8.0 ± 0.4	7.5 ± 0.4	6.7 ± 1.3	10.1 ± 0.9
GOT	232.2 ± 14.4^*^	292.9 ± 22.4	277.0 ± 1.5	295.9 ± 5.9^a^
GDH	19.6 ± 4.5	19.8 ± 0.6	16.3 ± 2.4	59.8 ± 2.5^a^

Values are mean ± S.E. (n = 3). Enzymatic activities are expressed as mU mg protein^−1^

(*) P < 0.05 vs. 12C and 12LC;

(#) P < 0.05 vs. 12LC;

(a) *P < 0.05 vs. 12LC*.

## Discussion

### Time course of metabolic capacities during the first 12 days of life

Common octopus paralarvae have a planktonic lifestyle that lasts until the first 30–60 days of life. Remaining yolk reserves provide energy substrates during the first few days post-hatching, however paralarvae immediately begin to actively capture live prey (Iglesias et al., 2007). This simultaneous use of endogenous reserves and exogenous food in cephalopods may last up to several days or weeks (Boletzky and Villanueva, 2014). Specifically, this type of feeding pattern has been shown in the common octopus to last approximately 5 days (Nande et al., 2017). Once the remaining yolk reserves are exhausted, paralarvae depend on active foraging involving episodes of burst swimming under anaerobic conditions (Baldwin, 1982). Our results indicated that both LDH and ODH provide energy to support such episodes of burst swimming during the first days of life. However, by day 9 ODH adopts a predominant role while LDH activity decreases. Therefore, the relative role of both systems in providing energy would be opposite to what has been observed in vertebrates (Lyzlova and Stefanov, 1991). This increase in anaerobic activity from day 9 could be due to the depletion of yolk reserves that would force paralarvae to increase their swimming activity for prey capture. Most mollusks only express ODH activity, and in the few cases where LDH is also present, ODH exhibits a higher level of activity (Regnouf and van Thoai, 1970; Gäde, 1980). Additionally, in a recent study on the enzymatic capacities of juvenile cuttlefish, no LDH activity was reported (Speers-Roesch et al., 2016). The coexistence of both pathways indicates that glycogen and arginine phosphate deposits in mantle muscle are used during burst swimming. Sustained PK activity would ensure the provision of pyruvate necessary for both reactions. Regarding the role of ODH, octopine and arginine are among the predominant free amino acids in cephalopod tissue (Lee, 1994). It has been reported that production of octopine might be advantageous in maintaining a low cytosolic redox ratio and, thus, glycolysis (Fields and Quinn, 1981) as well as intracellular osmotic pressure (Fields, 1983). Arginine has been reported to be the most abundant free amino acid in common octopus paralarvae (Villanueva et al., 2004), ensuring adequate levels of arginine phosphate and substrate for ODH. The importance of glycogen deposits in cephalopod muscle have been noted in the literature (O'Dor et al., 1984; Lee, 1994), and confirmed by LDH activity in the present study.

Once episodes of burst swimming ends, it is necessary for paralarvae to restore glycogen and arginine phosphate pools. However, data showing the importance of gluconeogenesis in cephalopods are scarce and contradictory. For example, where some studies indicate that gluconeogenesis occurs in several cephalopod tissues (Ballantyne et al., 1981; Fields and Hochachka, 1982; Hochachka and Fields, 1982), a recent study by Speers-Roesch et al. (2016) reported that *de novo* glucose synthesis is restricted to the digestive gland of juvenile cuttlefish, although the carbon source was unclear. In the present study, after an initial decrease at day 3, a significant increase occurred from day 6 post-hatching. Such increased activity, in parallel to that reported for anaerobic activity, might indicate an increased glycogen use or deposition. Based on the activity of transaminases and GyK in the present study, the substrate for glucose synthesis might be both amino acids and glycerol. It has been amply reported that amino acids are excellent gluconeogenic substrates that are incorporated into glycogen deposits of cephalopod mantle (Hochachka and Fields, 1982). Likewise, transaminases, mainly GOT, are among the enzymes that show higher specific activity in nature. The high GyK activity in hatched paralarvae seems consistent with a high hydrolysis of yolk lipids, resulting in glycerol incorporation into gluconeogenic/glycolytic pathways. Likewise, composition of yolk in cephalopods seems to include approximately 15% lipids (Caamal-Monsreal et al., 2015; Matozzo et al., 2015). Bouchaud and Galois (1990) reported that glyceryl monoesters are abundant in cuttlefish yolk and that probably plays an important role in energy metabolism. The slight decline in GyK activity until the sixth day post-hatching could be due to the depletion of yolk reserves while the subsequent increase may be attributed to greater availability of glycerol derived from catabolism of triacylglycerol present in live prey (Iglesias and Fuentes, 2014). Regarding the use of glycerol as a metabolic intermediate, overexpression of GAPDH has been reported in 4 day old common octopus paralarvae (Varó et al., 2017), which may be related to a higher rate of incorporation of G3P derived from GyK, to gluconeogenesis/glycolysis. Also, GAPDH would provide the NADH required for ODH and LDH reactions. Therefore, the present results seem to indicate that paralarvae develop metabolic capacities in a short period of time. A poor catabolic capacity would lead to a deficit of energy in the development of burst swimming which is necessary for capturing prey. These results also confirm the necessity of early metabolic capacity to fuel the capture of prey, even when yolk reserves have not been exhausted.

In addition to the energy demands imposed by the capture of prey supported by anaerobic metabolism, paralarvae need a supply of energy for growth, food processing, and maintenance of routine activity. These demands are typically supported by aerobic metabolism. In the present study, CS activity did not show significant changes during the ontogenic period analyzed, although the slight tendency to increase from third day could be related to growth and development processes that impose greater energy needs. GOT activity increased significantly in parallel to the observed changes in CS, indicating a positive correlation between both activities (*r* = 0.802, *P* < 0.001). This would ensure the provision of oxaloacetate necessary for the CS reaction. The data indicate that aspartate (a substrate of GOT) and glutamate, representing almost half of the non-essential amino acids in the body of cephalopods, are relevant (Villanueva et al., 2004). Protein catabolism in cephalopods has been widely accepted as the necessary fuel for aerobic metabolism, regardless of the body mass of the animal (Boucher-Rodoni and Mangold, 1985; Lee, 1994; Katsanevakis et al., 2005; Petza et al., 2006). This would imply a high requirement of this macronutrient in food (Houlihan et al., 1990; Navarro et al., 2014). Sustained GPT and GDH activities would also reflect active protein catabolism during the ontogenic period analyzed. However, the GPT/CS ratio (Figure 1) is higher in newly hatched paralarvae, possibly related to the predominance of the catabolism of protein, consisting of the major constituent of cephalopod yolk (Quintana et al., 2015). The reduced GPT/CS ratio from the third day onwards seems to indicate that part of the acetyl CoA incorporated to the CS reaction would come from a source different to amino acids. In this way, both HOAD activity and the HOAD/CS ratio (Figure 1) clearly show that since the sixth day forward a significant increase of fatty acid oxidation occurs. It has been widely accepted that cephalopods have a low capacity for lipid metabolism (Ballantyne et al., 1981; Storey and Storey, 1983; O'Dor and Webber, 1986; Lee, 1994; Hochachka, 1995), and as such, the requirement for lipid in food was very low (Seixas, 2009). However, Speers-Roesch et al. (2016) recently reported that cuttlefish efficiently use lipid-based fuels. The present study also shows that, at least during this phase, fatty acids are actively oxidized by common octopus paralarvae. The significant lower HOAD activity detected the first days of life would indicate a predominance of protein catabolism, as described above, probably due to the small proportion of lipid content in yolk and the higher protein content (Quintana et al., 2015).

### Influence of food composition on metabolic capacities and survival

The main result in this nutritional assay was that the paralarvae that were fed on LC60-enriched *Artemia* showed a 12% survival rate after 28 days, whereas less than 2% of the C group survived before that day. The results for SGR in the present study were similar to those reported previously for *O. vulgaris* paralarvae by Seixas et al. (2010) and Villanueva et al. (2004) using HUFA-enriched *Artemia* as live prey. Conversely, SGR results were lower than those previously found when LC60-enriched *Artemia* was provided (Garrido et al., 2017). However, SGR in both 12 and 28 day old paralarvae were higher in LC groups than in C groups. Thus, these data would indicate that food enrichment provided a beneficial effect on the welfare of paralarvae, although those paralarvae remaining after the 28 day sampling did not survive much longer.

Regarding the possible beneficial effect of food enrichment, in spite of the increased lipid content of LC60-enriched *Artemia*, oxidation rate of fatty acids did not increase in LC group, either at 12 or 28 days. This result may indicate that rather than being used as fuel, the higher amount of lipids could be contributing to the development of the nervous system, essential in such early stages (Nixon and Mangold, 1998). In this sense, the lipid-rich nervous system of *O. vulgaris* paralarvae hatchlings represents approximately one quarter of the animal's fresh weight (Packard and Albergoni, 1970). This may indicate the role of lipids for suitable growth during planktonic life. Increased G6PDH activity observed in 28LC groups would also contribute to such biosynthetic processes. Also, free glycerol derived from triacylglycerol hydrolysis might be used as substrate for glycolysis as indicates by the result of GyK, PK, and LDH at day 28. Increasing lipid levels in food would be not exerting the sparing effect of protein for growth amply reported for fish (Watanabe, 2002). According to Houlihan et al. (1990) and Moltschaniwskyj and Carter (2010), such a sparing effect of protein should give a high rate of protein synthesis and low protein degradation. However, 28 day old paralarvae showed a significant increase in GDH activity. It has been reported that the primary role of GDH in mantle muscle of squid is to regulate the catabolism of amino acids for energy production (Storey et al., 1978). The increased amino acid catabolism, registered in 28LC paralarvae, might be indicative of some kind of nutritional deficiency/imbalance.

In searching for a possible metabolic biomarker, GyK might be considered, since a significant increased activity was observed in both 12C and 28LC groups and a few days later an event of massive mortality took place. Although the role of G3P in cephalopods is controversial (Storey and Hochachka, 1975; Zammit and Newsholme, 1976), this metabolic intermediate can act as a carrier of cytosolic NADH to the mitochondrial electron transport chain. This shuttle activity is thought to coordinate glycolytic and mitochondrial metabolism in highly active tissues and is linked to ROS generation in mammals (Orr et al., 2012). Overproduction of ROS has been reported to exert detrimental effects on aquatic organisms (Abele et al., 2012). Hence, an increased G3P production might be linked to oxidative stress responsible for the low paralarvae survival.

## Conclusion

In summary, throughout the ontogenic period studied, an increase in anaerobic metabolism takes place, probably related to the increased dependence of paralarvae on exogenous food. Such activity is mainly supported by ODH activity, although LDH activity also contributes to the process. Regarding fuels that support aerobic metabolism, the use of amino acids was maintained and a significant increase in the rate of fatty acid oxidation was observed from the sixth day post-hatching. A low, albeit sustained, capacity for *de novo* synthesis of glucose from amino acids and glycerol was also observed. Feeding of larvae with phospholipid-enriched *Artemia* improved animal welfare, since there was a significant increase in the rate of survival and growth. Regardless of the composition of the food, GyK activity significantly increased a few days prior to a massive mortality event. This data may, in fact, indicate an imbalance in the redox state that could be responsible for the observed high mortality. Thus, GyK might be used as a nutritional and welfare biomarker.

## Author contributions

AM Analytical procedures, interpretation of the findings, and design, writing, and revision of the manuscript. GC Analysis, interpretation of the findings, and design, writing, and revision of the manuscript. MH Analysis and interpretation of the findings, and revision of the manuscript. DG and MM octopus paralarvae cultures and execution of the experiments, revision of the manuscript. EA design and execution of experiments, interpretation of results and revision of the manuscript. All authors read and approved the submitted manuscript.

### Conflict of interest statement

The authors declare that the research was conducted in the absence of any commercial or financial relationships that could be construed as a potential conflict of interest. The reviewer AT and handling Editor declared their shared affiliation, and the handling Editor states that the process nevertheless met the standards of a fair and objective review.
