# Supplementary material for: Time Course of Metabolic Capacities in Paralarvae of the Common Octopus, Octopus vulgaris, in the First Stages of Life. Searching Biomarkers of Nutritional Imbalance
Source: Front Physiol. 2017 Jun 16;8:427. doi: 10.3389/fphys.2017.00427 (PMC5473251; doi:10.3389/fphys.2017.00427)
Supplement: Supplementary file 1 [file Table1.docx]

Supplementary Material

Time course of metabolic capacities in paralarvae of the common octopus, *Octopus vulgaris*, in the first stages of life. Searching biomarkers of nutritional imbalance.

**Amalia E. Morales^1*^, Gabriel Cardenete^1^, M. Carmen Hidalgo^1^, Diego Garrido^2^, M. Virginia Martín^2^, Eduardo Almansa^2^**

*** Correspondence:** Amalia E. Morales; [amaenca@ugr.es](mailto:amaenca@ugr.es)

**Supplementary Table.** Assay conditions for enzyme activity monitoring.

| **ENZYME** | **REACTION MIXTURE** |
| --- | --- |
| Fructose 1,6-bisphosphatase  (FBPase; EC 3.1.3.11) | 50 mM imidazole-HCl buffer (pH 7.4), 5 mM MgCl_2_, 12 mM 2-mercaptoethanol, 0.5 mM NADP, 2 U/ml G6PDH, 2 U/ml PGI, 0.5 mM fructose 1,6-bisphosphate. |
| Glycerol kinase  (GyK; EC 2.7.1.30) | 50 mM imidazole-HCl buffer (pH 7.4), 0.75 mM NADH, 5 mM ATP, 5mM MgCl_2_, 10mM phosphoenolpyruvate, 4 U/ml PK, 4 U/ml LDH, 2.5 mM glycerol. |
| Pyruvate kinase  (PK; EC 2.7.1.40 | 50 mM imidazole-HCl buffer (pH 7.4), 100 mM KCl, 5 mM MgCl_2_, 1 mM ADP, 0.15 mM NADH, 2 U/ml LDH, 2 mM phosphoenolpyruvate. |
| Lactate dehydrogenase  (LDH; EC 1.1.1.27) | 50 mM imidazole-HCl buffer (pH 7.4), 0.15 mM NADH, 2.5 mM pyruvate. |
| Octopine dehydrogenase  (ODH; EC 1.5.1.11): | 50 mM imidazole-HCl buffer (pH 7.4), 0.15 mM NADH, 2.5 mM pyruvate, 5 mM L-arginine. |
| Glucose 6-phosphate dehydrogenase  (G6PDH; EC 1.1.1.49): | 50 mM imidazole-HCl buffer (pH 7.4), 5 mM MgCl_2_, 2 mM NADP, 1 mM glucose-6-phosphate. |
| Citrate synthase  (CS; EC 4.1.3.7) | 50 mM imidazole-HCl buffer (pH 8), 0.1 mM DTNB, 0.2 mM acetyl CoA, 0.2 mM oxaloacetic acid. |
| β-Hydroxyacyl CoA dehydrogenase  (HOAD; EC1.1.1.35) | 50 mM imidazole-HCl buffer (pH 8), 0.1 mM NADH, 0.2 mM acetoacetyl CoA. |
| Glutamate pyruvate transaminase  (GPT; EC 2.6.1.2) | 50 mM imidazole-HCl buffer (pH 7.4), 10 mM α-ketoglutarate, 0.2 mM NADH, 0.05 mM pyridoxal phosphate, 2 U/ml LDH, 25 mM L-alanine. |
| Glutamate oxaloacetate transaminase  (GOT; EC 2.6.1.1): | 50 mM imidazole-HCl buffer (pH 7.4),10 mM α-ketoglutarate, 0.3 mM NADH, 0.05 mM pyridoxal phosphate, 3 U/ml MDH, 25 mM L-aspartate. |
| Glutamate dehydrogenase  (GDH; EC 1.4.1.2): | 50 mM imidazole-HCl buffer (pH 8), 0.2 mM NADH, 1 mM ADP, 100 mM ammonium acetate, 10 mM α-ketoglutarate. |

**
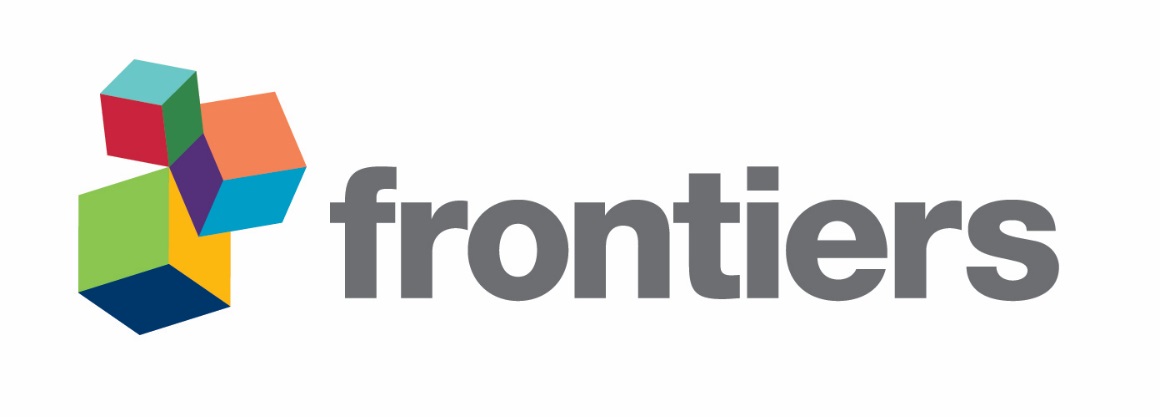
**
